# Non-suicidal self-injury function: prevalence in adolescents with depression and its associations with non-suicidal self-injury severity, duration and suicide

**DOI:** 10.3389/fpsyt.2023.1188327

**Published:** 2023-06-02

**Authors:** Ying Shen, Yingzi Hu, Yongjie Zhou, Xiwang Fan

**Affiliations:** ^1^Psychosomatic Medicine, The Third People’s Hospital of Ganzhou, Ganzhou, China; ^2^Department of Psychology, Fudan University, Shanghai, China; ^3^Clinical Research Center for Mental Disorders, Shanghai Pudong New Area Mental Health Center of Tongji University, Shanghai, China; ^4^Shenzhen Mental Health Center, Shenzhen Kangning Hospital, Shenzhen, China

**Keywords:** NSSI, self-harm function, adolescents, depression, suicide

## Abstract

**Background:**

Given that adolescents with depression are at the highest risk for non-suicidal self-injury (NSSI), a thorough understanding of their NSSI functions, as well as associations between functions and severe behavioral consequences, is essential for risk assessment and invention development.

**Methods:**

Adolescents with depression from 16 hospitals across China, for whom data was available regarding their NSSI function, frequency, number of methods used, time characteristics, and suicide history were included. Descriptive statistical analyses were performed to determine the prevalence of NSSI functions. Regression analyses were conducted to explore the relationship between NSSI functions and behavioral characteristics of NSSI and suicide attempts.

**Results:**

Affect regulation was the primary function of NSSI and followed by anti-dissociation in adolescents with depression. Females recognized automatic reinforcement functions more frequently than males, while males had a higher prevalence of social positive reinforcement functions. Automatic reinforce functions played the prominent role in associations between NSSI functions and all the severe behavioral consequences. Specifically, functions of anti-dissociation, affect regulation, and self-punishment were all associated with NSSI frequency, while higher levels of endorsements for anti-dissociation and self-punishment were linked to more NSSI methods, and greater level of endorsement for anti-dissociation was related to longer NSSI duration. Only the increase in endorsement of self-punishment was associated with a greater hazard of suicide attempts.

**Conclusion:**

The dominant functions of NSSI in adolescents with depression was automatic reinforcement, specifically affect regulation. And prevalence of NSSI function differed between males and females. Anti-dissociation and self-punishment seemed to be the most risky factors as they were linked to severe NSSI or suicide behaviors. More attention should be given to these functions in risk evaluation, and the targeted interventions should be developed accordingly in a timely manner.

## 1. Introduction

Non-suicidal self-injury (NSSI) is the “direct, deliberate destruction of one’s own body tissue in the absence of suicidal intent” (1). Adolescents are a vulnerable population for NSSI. Meta-analyses have revealed that the prevalence of NSSI is 17.2% among community adolescents (2) and 35%–80% among clinical adolescents (3). NSSI not only inflicts immediate physical pain and injury but also significantly elevates the risk of suicide attempts (4). Given its high prevalence and damage, NSSI has emerged as a significant public health issue worldwide (5). Nevertheless, avoidance of pain and injury is an instinct for living beings and is vital for their survival and reproduction. The compelling motives behind self-injury that transcend biological resistance are worth exploring.

NSSI functions refer to the motives or reinforcers of NSSI behavior (6). The four-function model (FFM) proposed by Nock is often considered the conceptual base of NSSI function, which classified NSSI functions into four major categories based on the source (intrapersonal or interpersonal) and nature (positive or negative) of reinforcement: automatic negative reinforcement (ANR), automatic positive reinforcement (APR), social negative reinforcement (SNR), and social positive reinforcement (SPR) (7). However, subsequent studies have not consistently supported the FFM and Klonsky (8) adapted it and proposed a two-factor model, which divided NSSI functions into intrapersonal function (linked to automatic reinforcement in the four-function model) and interpersonal function (linked to social reinforcement in the four-function model). Further research found that while the source of function (i.e., intrapersonal or interpersonal) can always be divided, the positive or negative nature of the function, especially ANR and APR in AR, could not always be clearly distinguished (9, 10). However, this differentiation was more evident in the social domain (11, 12). Thus, a three-factor structure has emerged. A similar study (13) based on Chinese adolescents in a clinical setting supported the three-factor model and divided NSSI functions into automatic reinforcement (AR), SNR, and SPR. Therefore, this study also adopted the three-factor model. AR stems from inner needs, such as affect regulation, anti-dissociation, or self-punishment. SNR refers to the effect of NSSI on avoiding interpersonal interactions or social activities. SPR considers NSSI as a means of interpersonal communication or influence, such as attracting attention or seeking understanding. A meta-analysis (14) found that intrapersonal functions, with reported prevalence rates ranging from 66% to 81%, are the most frequently reported and higher than social functions (from 33%–56%).

The knowledge of NSSI functions is essential for understanding the behavior, identifying potential treatment targets, and developing effective therapies (15). Despite its significance, NSSI function has received less attention than other characteristics of this behavior. A meta-analysis that examined the prevalence of NSSI functions found that results varied across studies, a potential reason for which was the heterogeneity of samples (14). It suggested that NSSI function may differ among various groups. For instance, a research has reported correlations between borderline personality disorder (BPD) symptoms and NSSI functions (16), indicating that NSSI functions may differ across various disorders. Thus, investigating NSSI function in a specific disorder may be necessary, particularly under the trend of precision medicine. Patients with depression were found to comprise the largest population of NSSI, and adolescents are the most vulnerable people for NSSI. The prevalence of NSSI in adolescents with depression was reported to reach 42.50% in China (17), a significant amount considering the country’s massive population and patients with mental disorders. Therefore, focusing on adolescents with depression would be a sensible approach to comprehensively understanding NSSI function and providing better medical services for this group.

Although NSSI is prevalent, its frequency and methods vary greatly among individuals (18). Given this high prevalence and diversity of NSSI, it is crucial to focus on the more severe aspects of NSSI for risk assessment and intervention in clinical practice. The severity of NSSI is usually measured by frequency and versatility, which refers to the number of methods used (19). Some studies suggest that automatic function (or intrapersonal function), rather than social function (or interpersonal function), is associated with more severe and enduring NSSI (20, 21). However, most of them have not investigated whether the association differs among specific functions (i.e., affect regulation, self-punishment, and anti-dissociation) within the automatic category. The value of such a conclusion is limited for both theoretical research and clinical work because automatic reinforcement function (AR) is just a broad and ambiguous category of NSSI function. So one aim of this study was to explore the relationship between specific AR functions and NSSI behavioral consequences, which may facilitate the accurate identification and effective treatment of individuals with severe NSSI, especially under the circumstance of commonly relative scarcity of medical resource.

Although NSSI is not primarily driven by suicidal intentions, it is closely associated with suicide. Numerous studies have confirmed the high comorbidity between NSSI and suicide such as (22). However little is known about the mechanisms underlying this association. Most studies have focused on the behavioral characteristics of NSSI and identified that the frequency and versatility of NSSI are the second strongest predictors of suicide attempts, just behind suicidal thoughts (23). This suggests that determining the characteristics of NSSI may be a promising avenue for estimating latent suicide risk, especially when suicide plans or attempts are private or sudden. Moreover, studies have found that NSSI and suicide may partly overlap in motivation (24), implying that individuals engaging in NSSI under certain functions are more likely to simultaneously develop or engage in suicidal behavior. Therefore, it would be worthwhile to explore the association between NSSI functions and suicide attempts, particularly in adolescents with depression, who are commonly considered as the most vulnerable and main group of NSSI and suicide behaviors (25).

This study has three main objectives: first, we aim to describe the prevalence of NSSI functions in adolescents with depression based on a large sample. Secondly, we intend to investigate the association between NSSI functions and its frequency, versatility, and duration. Based on previous research, we hypothesize that AR will play a prominent role. And in the subsequent step, the exploration of which specific AR functions are correlative to severe behaviour consequences will be conducted. Finally, we aim to investigate the potential association between NSSI functions and co-occurring suicide attempts.

## 2. Methods

### 2.1. Sample and procedure

Teenage patients were recruited from 16 hospitals’ psychiatric outpatient clinics or inpatient departments in nine provinces of China using convenience sampling between December 2020 and December 2021. Inclusion criteria for the study were as follows: (1) adolescents between the ages of 12–18 years old; (2) a diagnosis of depressive episode made by experienced clinical psychiatrists in accordance with the ICD-10; (3) at least one instance of NSSI behavior in the past year; and (4) having finished primary education. The exclusion criteria were as follows: (1) experiencing acute episode of obvious psychotic symptoms, and (2) presenting with cognitive or intellectual impairments.

This study was conducted in accordance with the Declaration of Helsinki and approved by the Ethics Committee of the Institutional Review Board (IRB) of Shenzhen Kangning Hospital (020-k021-02). Written informed consent was obtained from the participants and their legal guardians.

Participants completed the questionnaire independently, with a trained research assistant available to answer any questions they might have had. To ensure data quality, research assistants would check the questionnaires after completion, and any unusual answers would be confirmed.

A total of 1,101 participants were included, with 199 (18.07%) males and 902 (81.93%) females, and a mean age of 14.7 (SD = 1.63). Nearly half of the participants (44.96%) had experienced their first episode of depression. More than half (64.12%) lived in cities. Only about a quarter (25.16%) were only-child, whereas 74.84% had siblings. Nearly 20% had experienced being left-behind, which is a common social phenomenon in China, especially in the underdeveloped regions. It refers to children who are left behind in their hometowns while their parents work and earn money in larger cities. In this study, participants who had experienced being left-behind for over 1 year were classified as left-behind children. Most children (77.38%) had parents in marriage, while 19.62% had parents divorced or separated, and 3% had one or both deceased. In terms of educational status, the majority of adolescents with depression (79.75%) were enrolled in school, 18.17% were suspended, and 2.09% had dropped out of school. The demographic and clinical information of the participants has been shown in Table 1.

**Table 1:** Demographic and clinical characteristics.

	% or *M* (SD)
Gender	
Male	18.07%
Female	81.93%
Age	14.7 (1.63)
Course of disease	
First-episode	44.96%
<6 months	21.34%
6 months–1 year	11.26%
1 year-2 years	12.08%
≥2 years	9.81%
Residence	
Urban	64.12%
Rural	35.88%
Family structure	
Only-child	25.16%
Multi-child	74.84%
Left-behind experience	
Yes	19.26%
No	80.74%
Marital status of parents	
In marriage	77.38%
Divorced or separated	19.62%
One or both deceased	3.00%
Education status	
At school	79.75%
Suspend school	18.17%
Quit school	2.09%

### 2.2. Measures

#### 2.2.1. Demographics

In the present study, participants were required to provide their basic personal information in a questionnaire, which included demographic characteristics such as gender, age, disease course, place of residence, family components, experience of being left behind, parents’ marital status, and education status.

#### 2.2.2. NSSI characteristics

NSSI behavior and function characteristics are often assessed by the functional assessment of self-mutilation questionnaire (FASM, 26), which has been demonstrated to possess good psychometric properties in both normal populations (27, 28) and clinical samples (29) across various countries. Qu et al. (13) translated and localized the scale and produced the Chinese version of FASM (C-FASM). In this study, all NSSI variables were measured using the C-FASM, which consists of three main parts. The first part evaluates 11 self-harm methods and their frequency in the past year. The second part inquires about age of the first NSSI episode, hesitation before action (from “not considered” to “greater than 1 week”), and degree of physical pain during NSSI (from “no pain” to “severe pain”). The last part measures the endorsement of 15 functions using a four-point Likert scale (from “never” to “often”), which can be categorized into three factors: AR, SNR, and SPR. AR includes three main individual functions: affect regulation (“to stop bad feelings” and “to feel relaxed”), anti-dissociation (“to relieve feeling numb or empty” and “to feeling something, even if it was pain”), and self-punishment (“to punish yourself”). The values of affect regulation and anti-dissociation function are the mean of the two items describing them, and the value of self-punishment function is the score of “to punish yourself” item. SNR primarily refers to avoiding social activities (e.g., “to avoid doing something unpleasant you do not want”) or interpersonal interactions (“to avoid being with people”). SPR focuses on seeking positive interpersonal feedback (e.g., “to get your parents to understand or notice you”). All the three factors demonstrated acceptable internal consistency in this sample, AR-*α* = 0.71, SNR-*α* = 0.72, and SPR-*α* = 0.87. NSSI behavior characteristics, including frequency and methods used, should be reported based on the fact in the past year.

#### 2.2.3. Suicide attempts

Participants were queried regarding their history of suicidal thoughts or attempts, and if a suicidal behavior had occurred, the specific time should be noted. To investigate the co-occurrence of NSSI and suicide behaviour, only suicide attempts within the past year were considered to correspond with NSSI.

### 2.3. Data analysis

The measurement of NSSI versatility was based on the number of methods reported by participants, and the sum of NSSI frequency under each method was used to calculate the total NSSI behavior frequency. NSSI duration was defined as the difference between the current and first NSSI age and there were 64 cases with missing data in the first NSSI age were excluded from duration calculation and related data analyses. The scores of the AR, SNR, and SPR subscales were calculated as the mean of their respective items due to differences in the number of items included. The skewness of all metric variables (except NSSI frequency) was within an acceptable range [<2 (30),]. To address non-normality in NSSI frequency, a natural logarithmic transformation was applied, resulting in approximately normal distribution of the transformed frequency data for use in all relevant analyses.

Descriptive statistics, including percentages, means, and standard deviations, were used to provide a concise report of the basic characteristics of NSSI in this sample. The prevalence of NSSI functions was determined through the calculation of endorsement rates for each function. Gender differences were examined using Mann–Whitney *U* tests for every single function and *t*-tests for function categories. Linear regression was conducted to analyze NSSI frequency. As for the two count variables, negative binomial regression was used for NSSI versatility and Poisson regression was for NSSI duration. It was due to the overdispersion observed in NSSI versatility (but not in NSSI duration), with its variance (6.40) exceeding the mean (4.30) and the dispersion parameter (1.11) was over 1. And binary logistic regression was utilized to explore suicide attempts (yes/no). In all regression analyses, the three function categories were first entered to construct category models. And then, models of the individual AR functions were taken for further examinations. Control variables, including age, gender, and hospital site, were included in all regression analyses to eliminate potential confounding effects. All analyses were performed using SPSS version 26.0. Statistical significance was set at a two-tailed *p* < 0.05 (as the default value), and Bonferroni correction may be implemented in the event of necessity.

## 3. Results

### 3.1. NSSI characteristics

According to the criteria of Muehlenkamp et al. (31), patients were stratified into three groups based on the NSSI frequency they reported: low-frequency (<5), medium-frequency (5–25), and high-frequency (≥25), corresponding to proportions of 9.72%, 28.07%, and 62.22%, respectively. The mean number of methods employed was 4.30 (SD = 2.53), with the most common being “cut or crave on skin” (87.83%) and “hit self” (52.59%). The mean age of onset for NSSI was 13.31 (SD = 1.71) years, with an average duration of 1.41 (SD = 1.21) years. More than half of adolescents with depression (51.50%) engaged in the behavior without hesitation, while some (26.61%) hesitated for only a few minutes, and the majority (88.28%) did not hesitate for more than 1 h. Regarding the physical pain experienced during NSSI, more than half (51.23%) reported mild pain, nearly 30% did not feel any pain, 17.71% reported moderate pain, and only 2.45% reported severe pain.

### 3.2. Prevalence of NSSI functions

The most frequently reported functions were “to stop bad feelings” and “to feel relaxed,” followed by “to relieve feeling numb or empty,” all of which fell under the AR category. The most commonly reported functions of SNR were “to avoid doing something unpleasant you do not want” and “to avoid being with people.” The function of “to let others know how desperate you were” and “to get your parents to understand or notice you” received the highest endorsement in SPR. Further elaboration on the prevalence of NSSI functions can be observed in Table 2.

**Table 2:** Prevalence of NSSI functions.

Functions	Total	Male	Female	Total *M* (SD)	*t* or *z* (*p*)	Cohen’s *d*
**AR**				1.76 (0.77)	**4.54 (0.000)**	0.36
To stop bad feelings	85.38%	78.89%	86.81%		**3.66 (0.000)**	0.30
To feel relaxed	81.65%	74.37%	83.26%		**3.76 (0.000)**	0.30
To relieve feeling numb or empty	80.47%	76.88%	81.26%		1.40 (0.162)	0.11
To punish yourself	75.66%	65.33%	77.94%		**4.38 (0.000)**	0.35
To feel something, even if it was pain	73.66%	70.85%	74.28%		1.89 (0.059)	0.15
**SNR**				0.79 (0.77)	0.08 (0.938)	0.01
To avoid doing something unpleasant you do not want	52.41%	54.77%	51.88%		0.76 (0.446)	0.05
To avoid being with people	47.59%	47.24%	47.67%		0.3 (0.764)	0.03
To avoid school, work, or other activities	40.42%	41.71%	40.13%		0.32 (0.750)	0.02
To avoid punishment or paying the consequences	32.97%	34.17%	32.71%		0.09 (0.929)	0.06
**SPR**				0.69 (0.77)	**−3.05 (0.002)**	0.24
To let others know how desperate you were	46.41%	52.26%	45.12%		−1.87 (0.061)	0.14
To get your parents to understand or notice you	40.69%	43.72%	40.02%		−1.12 (0.264)	0.09
To get help	38.24%	39.20%	38.03%		−0.09 (0.927)	0.02
To get attention	37.97%	48.74%	35.59%		**−3.54 (0.000)**	0.26
To try to get a reaction from someone, even negative	36.88%	53.27%	33.26%		**−5.38 (0.000)**	0.40
To receive more attention from your parents or friends	35.79%	47.24%	33.26%		**−3.99 (0.000)**	0.30

*p* after Bonferroni correction = 0.05/18 = 0.0028. Bold = significant after Bonferroni correction.

Only 15.99% of participants endorsed a single function category, which was AR (15.44%) in most cases. Furthermore, 29.43% of participants endorsed two function categories, including AR + SNR (17.08%) and AR + SPR (11.99%). More than half of the participants (54.59%) endorsed all three function categories.

A significant difference was observed in the degree of recognition of the three function categories (*F* = 650.73, *p* = 0.000***, *η_p_*^2^ = 0.28), with *post hoc* analysis indicating that all pairs significantly differed from each other (*p* = 0.000***). And the results indicated that the most commonly endorsed function category of NSSI in adolescents with depression was AR, followed by SNR, and then SPR.

However, there were some differences between males and females in the prevalence of NSSI functions. Specifically, females endorsed AR > SNR > SPR (*F* = 611.07, *p* = 0.000***, *η_p_*^2^ = 0.31; *p* = 0.000***). Males endorsed AR > SPR and SNR (*F* = 59.47, *p* = 0.000***, *η_p_*^2^ = 0.17) and no significant difference was noted between SPR and SNR (*p* = 0.54). Females endorsed each function of AR at a higher rate than males, with differences ranging from 1.34% to 12.61%. The mean score of AR category in females was significantly higher than in males, with a moderate effect size. The Mann–Whitney U test showed that females had higher endorsement in the function of “to punish yourself,” “to feel relaxed,” and “to stop bad feelings” than males. Males endorsed each function of SPR higher than females, especially “to try to get a reaction from someone, even negative,” for which the rate was 20.01% lower in females, indicating a significant difference. This was where the greatest gender difference was observed. Males also gave more endorsement to the function of “to receive more attention from your parents or friends” and “to get attention” in SPR than females, and the score of SPR category was significantly higher in females than in males. However, no significant difference existed in SPR between males and females. Age did not correlate with social function and had a significant but weak negative correlation with AR (*r* = −0.07, *p* = 0.018*).

### 3.3. Functions and the severity and duration of NSSI

Age, gender, hospital site, and the three function categories were inputted simultaneously (model 1) to conduct the linear regression analysis of NSSI frequency. Result revealed that except age, only AR was significant after the Bonferroni correction. Although the *p***-**value of SNR was below 0.05, it did not meet the Bonferroni correction and the beta weight was small. *R*^2^ of the model was 0.23, which mean that the model accounted for approximately 23% variation of NSSI frequency. In Model 2, which focused on the three specific AR functions, affect regulation, anti-dissociation, and self-punishment were all independently associated with NSSI frequency, see Table 3.

**Table 3:** Regressions of NSSI functions and the severity and duration.

	NSSI frequency	NSSI versatility	NSSI duration
	*β* (*p*)	OR (*p*)	OR (*p*)
Model 1			
Age	**−0.12 (0.000)**	0.95 (0.009)	**1.18 (0.000)**
Gender^a^	0.01 (0.651)	1.02 (0.810)	1.18(0.046)
Hospital cite	0.03 (0.345)	1.01 (0.383)	1.01 (0.144)
AR	**0.43 (0.000)**	**1.41 (0.000)**	**1.22 (0.000)**
SNR	0.07 (0.016)	1.07 (0.187)	1.01 (0.839)
SPR	−0.03 (0.250)	1.00 (0.928)	1.05 (0.259)
Model 2			
Age	**−0.13 (0.000)**	**0.94 (0.005)**	**1.18 (0.000)**
Gender^a^	0.00 (0.998)	1.02 (0.799)	1.18 (0.047)
Hospital cite	0.03 (0.287)	1.01 (0.332)	1.01 (0.145)
Affect regulation	**0.18 (0.000)**	1.13 (0.012)	1.06 (0.167)
Anti-dissociation	**0.25 (0.000)**	**1.15 (0.000)**	**1.11 (0.003)**
Self-punishment	**0.14 (0.000)**	**1.13 (0.000)**	1.06 (0.031)

*β*(*p*), standardized regression coefficients with *p*-value. *p after* Bonferroni correction = 0.05/8 = 0.00625. Bold = significant after Bonferroni correction.

a Female

In the negative binomial regression analysis for NSSI versatility, another indicator of NSSI severity, AR was also the only significant function category. And in model 2, only anti-dissociation and self-punishment survived the Bonferroni correction.

The same procedure was conducted for the Poisson regression of NSSI duration, and likewise only AR was significantly associated with enduring NSSI. However, only anti-dissociation survived in model 2, while self-punishment did not meet the Bonferroni correction and affect regulation failed to reach statistical significance totally.

### 3.4. NSSI functions and suicide attempt

Among adolescents with depression and NSSI, the vast majority (97.09%) reported having suicidal thoughts, and nearly half (49.59%) reported lifetime suicide attempts. Additionally, over 30% experienced both NSSI and suicide attempts in the recent 1 year.

Through binary logistic regression analysis, only affect regulation (AR) was found to be associated with an increased risk of suicide attempts. Specifically, an increase in the endorsement of self-punishment was independently relevant to concurrent suicide attempts (refer to Table 4 for detailed results).

**Table 4:** Regressions of NSSI functions and suicide attempt.

	*OR*	*p*	95% CI	99.375% CI	*χ*^2^
**Model 1**					**25.03**
Age	0.94	0.116	(0.87, 1.02)	(0.84, 1.05)	
Gender^a^	0.99	0.958	(0.70, 1.40)	(0.61, 1.60)	
Hospital cite	0.96	0.014	(0.93, 0.99)	(0.92, 1.00)	
AR	**1.35**	0.001	(1.13, 1.63)	(1.05, 1.75)	
SNR	0.90	0.267	(0.74, 1.09)	(0.69, 1.17)	
SPR	1.19	0.056	(1.00, 1.43)	(0.93, 1.53)	
**Model 2**					**25.67**
Age	0.94	0.125	(0.87, 1.02)	(0.84, 1.05)	
Gender^a^	1.04	0.819	(0.74, 1.47)	(0.65, 1.68)	
Hospital cite	0.96	0.021	(0.94, 0.99)	(0.92, 1.01)	
Affect regulation	1.11	0.273	(0.92, 1.34)	(0.86, 1.44)	
Anti-dissociation	1.03	0.661	(0.89, 1.20)	(0.84, 1.27)	
Self-punishment	**1.21**	0.002	(1.07, 1.36)	(1.02, 1.43)	

*p* after Bonferroni correction = 0.05/8 = 0.00625. Bold = significant after Bonferroni correction.

a Female.

## 4. Discussion

This study sought to investigate the primary functions of NSSI in adolescents with depression, and ascertain the risky functions associated with severe behavioral consequences. The main findings of the study are as follows: (1) affect regulation was the primary NSSI function in adolescents with depression, followed by an elevated function of anti-dissociation observed in this group. And gender difference observed in the prevalence of NSSI function was that Females recognized AR more frequently than males, while males had a higher prevalence of SPR. (2) AR played the prominent role in associations between NSSI functions and severe behavioral consequences. And within AR, functions of anti-dissociation, affect regulation, and self-punishment were all positively correlated with frequency of NSSI. And higher levels of endorsements for anti-dissociation and self-punishment were linked to more NSSI versatility. Only greater levels of endorsement for anti-dissociation were found to be linked to longer NSSI duration. (3) AR was also associated with a higher risk of suicide attempts, and only the increase in self-punishment endorsement was independently related to a greater hazard of suicide attempts. Case-by-case discussions are presented below.

### 4.1. Prevalence of NSSI functions and gender difference

The most prevalent function was affect regulation (“to stop bad feelings” and “to feel relaxed”), which is in line with the prevailing view that NSSI is primarily used to alleviate negative emotions, reduce arousal levels, and simultaneously bring about positive feelings, such as relaxation or calmness (8, 32). The nature of NSSI, as a maladaptive emotion regulation strategy, has also been demonstrated in adolescents with depression (33). Anti-dissociation (e.g., “to relieve feeling numb or empty”) was the second most common function, which was somewhat at odds with general findings. Meta-analyses (14, 34) and a study on BPD adolescents (16) reported that anti-dissociation was the relatively less endorsed function. However, our sample revealed a higher prevalence of it, which may be attributed to the association of depression with feelings of numbness and emptiness. NSSI can provide a person with physical and emotional sensations that facilitate a sense of authenticity and self-awareness, which could be the compensation and particularly reinforce the recognition of the anti-dissociation function in depression (6). In addition, a study using pathway analysis found that the relationship between post-traumatic stress, depression, and self-harm is mediated through dissociation, suggesting that anti-dissociation may be the crucial factor in the high co-occurrence of NSSI and depression (35).

Pronounced trait of avoidance was observed in NSSI function. Consistent with the hypothesis proposed by Nock and Prinstein (7), NSSI functions involve both decrease in negative outcomes and increase in positive outcomes, which often occur simultaneously and intertwine (36, 37). However, our results revealed a greater endorsement of negative aspects compared to positive ones (e.g., “to relieve feeling numb or empty” exceeded “to feel something, even if it was pain”). Similar findings have been reported in other studies (28, 38). These results suggested that avoiding unwanted internal states may be more desirable than inducing positive states or that negative reinforcement may be more potent in NSSI, which supports the experience avoidance model of NSSI (39). Individuals who engage in NSSI may be experiencing severe psychological distress and require urgent professional assistance.

The majority of adolescents with depression in this study had both automatic and social functions, and more than half recognized all three functional categories, which demonstrates the cross-functionality of NSSI. AR was consistently more prevalent than SNR and SPR, regardless of gender. However, females endorsed SNR more than SPR, while this difference was not observed in males. Males had significantly higher SPR scores than females, indicating a greater likelihood of self-harm to gain positive social feedback, such as attention or response. One theoretical perspective suggests that NSSI serves as a powerful form of communication to signal psychological distress and avoid abandonment or seek understanding (40, 41). (42) conducted a diary study and found that disclosing NSSI behaviors significantly increased perceived social support. NSSI may be a means of influencing the environment, reflecting the adaptability of this behavior, particularly in males who may experience difficulty expressing their emotions or needs. Additionally, we found that females had significantly higher AR scores than males, who might be more likely to engage in NSSI as a means of self-punishment or affect regulation. This finding is consistent with previous research (43) and may be attributed to the greater prevalence of severe internal problems among females (44).

### 4.2. Individual automatic functions and the severity, duration of NSSI

AR was associated with more severe and persistent NSSI in this study, which is in line with the results of previous studies (42, 46). Although affect regulation was the most popular function, it no longer played the dominant role in the association between NSSI function and worse behavioral outcomes. Our study further confirmed that the three individual automatic functions, anti-dissociation, affect regulation, and self-punishment were associated with NSSI frequency, and anti-dissociation together with self-punishment were associated with NSSI versatility. Only anti-dissociation was independently associated with NSSI duration. Moreover, anti-dissociation beyond affect regulation showed the strongest overall association, which has also been partially reflected in Reinhardt’s study (46) that reported correlation coefficients between them. And our study further confirmed these relationships through regression analysis.

Our findings partly confirmed and refined the findings of a preliminary study by Yen et al. (29) that it’s anti-dissociation rather than affect regulation that was involved in the maintenance of NSSI. This can be understood in terms of stability. A follow-up study (47) found that affect regulation was the most variable function. Negative emotions easily subside as the internal or external environment improves, at which point there appears to be no reason to continue NSSI.

### 4.3. Individual automatic functions and suicide attempt

AR was identified as a significant factor associated with suicide attempts, consistent with prior research (42). However, our study revealed that only an increased in the self-punishment function was independently associated with an elevated risk of suicide attempt. Chapman et al. (39) posited that NSSI as a form of self-punishment could alleviate negative self-cognition, shame, and self-hatred. And it has been supported by the findings of experimental studies (48). However, unlike other functions, self-harm motivated by self-punishment implicates anger and aggression directed toward oneself. It can run through the continuum from NSSI to suicidal self-injury and escalate to the latter (49). That is, when the motivation to punish oneself expands to some extent, the most extreme form, suicide, may be the chosen course of action.

It is generally believed that intense psychological distress and despair are the primary drivers of suicide (50). Surprisingly, we did not observe a role of affect regulation in the co-occurrence of NSSI and suicide. This could be because affect regulation was already effectively achieved through NSSI. As reported by Saraff et al. (21), the majority of participants (94%) recognized the role of NSSI in emotional relief. Brausch and Muehlenkamp (47) also found that affect regulation was the most effective function in self-reporting. These findings demonstrate the value of NSSI as a coping strategy, as it may mitigate the risk of more serious consequences, even if doing so incurs costs.

### 4.4. Clinical implications

Our findings highlight the significance of conducting a comprehensive assessment of NSSI function. A high endorsement of automatic functions could indicate severe NSSI and underlying suicide attempts, which necessitate close monitoring and intensive treatment. In addition, given the multifacetedness NSSI function, mental health practitioners and clinicians should comprehend the NSSI functions that patients endorse and devise appropriate treatment plans. Currently, the primary therapy for NSSI is dialectical behavioral therapy [DBT (51),], which concentrates on affect regulation and enhancement of emotion regulation ability and distress tolerance. However, given our findings that functions of anti-dissociation and self-punishment might be the more important risky factors, we recommend that treatments targeting dissociation and negative self-cognition should be considered, such as behavioral activation (52), mindfulness (53), and cognitive behavioral therapy (CBT), particularly in individuals experiencing depression.

### 4.5. Limitations

This study has some limitations that should be considered when interpreting the findings. Firstly, the data were cross-sectional, which means that only associations between NSSI functions and behavioral outcomes were examined, and directionality and causality could not be clarified. Future longitudinal studies should be considered to verify these findings and explore the predictive role of NSSI functions in treatment effects or the transition from NSSI to suicide. Secondly, participants were only asked to report on NSSI that occurred within the past year. The lifetime data was unknown, which represents the overall level and may potentially impacting the results. Thirdly, self-reported data may be subject and exist bias. The use of ecological momentary assessment techniques could be considered to obtain more objective and impartial behavioral and physiological data. Finally, NSSI function is not necessarily equivalent to reinforcement, as sometimes the functions are not effectively satisfied. We suggest that future studies could include measures of function validity to gain a deeper understanding of associations between NSSI functions and behavioral outcomes from these subtle but essential perspectives.

### 4.6. Conclusion

In this study, we shed light on the significance of understanding the functions of NSSI in identifying prevalent motivations and potential risk factors associated with severe behaviors. Based on a large sample, we identified affect regulation as the most prevalent function, and found an increase in the endorsement of anti-dissociation function in adolescents with depression. We also found gender difference that females recognized AR more frequently than males, while males had a higher prevalence of SPR. AR was not only prevalent but also the risky function category linked to severe outcomes. However, function of affect regulation no longer played the primary role in associations between NSSI function and behavioral consequences as it did in the prevalence aspect. Instead, anti-dissociation and self-punishment seemed to be more risky factors. Indicators of these functions should be given more attention to risk evaluation. We recommend that more attention should be given to these functions in risk evaluation of NSSI, and the targeted intervention or treatment should be developed accordingly in a timely manner.

## Data availability statement

The raw data supporting the conclusions of this article will be made available by the authors, without undue reservation.

## Ethics statement

The studies involving human participants were reviewed and approved by the Ethics Committee of the Institutional Review Board (IRB) of Shenzhen Kangning Hospital (020-k021-02). Written informed consent to participate in this study was provided by the participants’ legal guardian/next of kin.

## Author contributions

YS and YZ: data collection. YS and YH: conceptualization, design and methodology. YH: data analysis and draft writing. YZ and XF: supervision, verification and editing. YZ and XF: project administration and funding acquisition. All authors contributed to the article and approved the submitted version.

## Funding

This study was supported by the Medical discipline Construction Project of Pudong Health Committee of Shanghai: (Grant No. PWZzb2022-09), Medical discipline Construction Project of Pudong Health Committee of Shanghai: (Grant No. PWYgy2021-02), Sanming Project of Medicine in Shenzhen (No. SZSM202011014), and Shenzhen Fund for Guangdong Provincial High-level Clinical Key Specialties (No. SZGSP013).

## Conflict of interest

The authors declare that the research was conducted in the absence of any commercial or financial relationships that could be construed as a potential conflict of interest.

## Publisher’s note

All claims expressed in this article are solely those of the authors and do not necessarily represent those of their affiliated organizations, or those of the publisher, the editors and the reviewers. Any product that may be evaluated in this article, or claim that may be made by its manufacturer, is not guaranteed or endorsed by the publisher.

## References

[1] Nock MK Favazza AR. Nonsuicidal self-injury: Definition and classification. In: MK Nock editor. Understanding nonsuicidal self-injury: Origins, assessment, and treatment. American Psychological Association. (2009) 9–18.

[2] Swannell SV Martin GE Page A Hasking P St John NJ. Prevalence of nonsuicidal self-injury in nonclinical samples: systematic review, meta-analysis and meta-regression. Suicide Life Threat Behav. (2014) 44:273–303. doi: 10.1111/sltb.12070, PMID: 24422986

[3] Glenn CR Klonsky ED. Nonsuicidal self-injury disorder: an empirical investigation in adolescent psychiatric patients. J Clin Child Adolesc Psychol. (2013) 42:496–507. doi: 10.1080/15374416.2013.794699 23682597 PMC4433043

[4] Ribeiro JD Franklin JC Fox KR Bentley KH Kleiman EM Chang BP. Self-injurious thoughts and behaviors as risk factors for future suicide ideation, attempts, and death: a meta-analysis of longitudinal studies. Psychol Med. (2016) 46:225–36. doi: 10.1017/S0033291715001804, PMID: 26370729 PMC4774896

[5] Mumme TA Mildred H Knight T. How do people stop non-suicidal self-injury? A systematic review. Arch Suicide Res. (2017) 21:470–89. doi: 10.1080/13811118.2016.1222319, PMID: 27610697

[6] Klonsky ED Muehlenkamp JJ. Self-injury: a research review for the practitioner. J Clin Psychol. (2007) 63:1045–56. doi: 10.1002/jclp.20412, PMID: 17932985

[7] Nock MK Prinstein MJ. A functional approach to the assessment of self-mutilative behavior. J Consult Clin Psychol. (2004) 72:885–90. doi: 10.1037/0022-006X.72.5.885, PMID: 15482046

[8] Klonsky ED. The functions of self-injury in young adults who cut themselves: clarifying the evidence for affect-regulation. Psychiatry Res. (2009) 166:260–8. doi: 10.1016/j.psychres.2008.02.008 19275962 PMC2723954

[9] Kaess M Parzer P Mattern M Plener PL Bifulco A Resch F. Adverse childhood experiences and their impact on frequency, severity, and the individual function of nonsuicidal self-injury in youth. Psychiatry Res. (2013) 206:265–72. doi: 10.1016/j.psychres.2012.10.012, PMID: 23159195

[10] Leong CH Wu AM Poon MM. Measurement of perceived functions of non-suicidal self-injury for Chinese adolescents. Arch Suicide Res. (2014) 18:193–212. doi: 10.1080/13811118.2013.824828 24568552

[11] Bentley KH Nock MK Barlow DH. The four-function model of nonsuicidal self-injury. Clin Psychol Sci. (2014) 2:638–56. doi: 10.1177/2167702613514563

[12] Dahlstrom O Zetterqvist M Lundh LG Svedin CG. Functions of nonsuicidal self-injury: exploratory and confirmatory factor analyses in a large community sample of adolescents. Psychol Assess. (2015) 27:302–13. doi: 10.1037/pas0000034, PMID: 25558962

[13] Qu D Wang Y Zhang Z Meng L Zhu F Zheng T. Psychometric properties of the Chinese version of the functional assessment of self-mutilation (FASM) in Chinese clinical adolescents. Front Psych. (2021) 12:755857. doi: 10.3389/fpsyt.2021.755857, PMID: 35153848 PMC8826685

[14] Taylor PJ Jomar K Dhingra K Forrester R Shahmalak U Dickson JM. A meta-analysis of the prevalence of different functions of non-suicidal self-injury. J Affect Disord. (2018) 227:759–69. doi: 10.1016/j.jad.2017.11.073, PMID: 29689691

[15] Washburn JJ Richardt SL Styer DM Gebhardt M Juzwin KR Yourek A. Psychotherapeutic approaches to non-suicidal self-injury in adolescents. Child Adolesc Psychiatry Ment Health. (2012) 6:14. doi: 10.1186/1753-2000-6-14, PMID: 22463499 PMC3782878

[16] Sadeh N Londahl-Shaller EA Piatigorsky A Fordwood S Stuart BK McNiel DE. Functions of non-suicidal self-injury in adolescents and young adults with borderline personality disorder symptoms. Psychiatry Res. (2014) 216:217–22. doi: 10.1016/j.psychres.2014.02.018, PMID: 24594204

[17] Shao C Wang X Ma Q Zhao Y Yun X. Analysis of risk factors of non-suicidal self-harm behavior in adolescents with depression. Ann Palliat Med. (2021) 10:9607–13. doi: 10.21037/apm-21-1951 34628886

[18] Klonsky ED Olino TM. Identifying clinically distinct subgroups of self-injurers among young adults: a latent class analysis. J Consult Clin Psychol. (2008) 76:22–7. doi: 10.1037/0022-006X.76.1.22, PMID: 18229979

[19] Ammerman BA Jacobucci R Turner BJ Dixon-Gordon KL McCloskey MS. Quantifying the importance of lifetime frequency versus number of methods in conceptualizing nonsuicidal self-injury severity. Psychol Violence. (2020) 10:442–51. doi: 10.1037/vio0000263

[20] Halpin SA Duffy NM. Predictors of non-suicidal self-injury cessation in adults who self-injured during adolescence. J Affect Disord Rep. (2020) 1:100017. doi: 10.1016/j.jadr.2020.100017

[21] Saraff PD Trujillo N Pepper CM. Functions, consequences, and frequency of non-suicidal self-injury. Psychiatry Q. (2015) 86:385–93. doi: 10.1007/s11126-015-9338-6 25597028

[22] Cheung YT Wong PW Lee AM Lam TH Fan YS Yip PS. Non-suicidal self-injury and suicidal behavior: prevalence, co-occurrence, and correlates of suicide among adolescents in Hong Kong. Soc Psychiatry Psychiatr Epidemiol. (2013) 48:1133–44. doi: 10.1007/s00127-012-0640-4, PMID: 23262815

[23] Victor SE Klonsky ED. Correlates of suicide attempts among self-injurers: a meta-analysis. Clin Psychol Rev. (2014) 34:282–97. doi: 10.1016/j.cpr.2014.03.005 24742496

[24] Brown MZ Comtois KA Linehan MM. Reasons for suicide attempts and nonsuicidal self-injury in women with borderline personality disorder. J Abnorm Psychol. (2002) 111:198–202. doi: 10.1037//0021-843x.111.1.198, PMID: 11866174

[25] Ougrin D Latif S. Specific psychological treatment versus treatment as usual in adolescents with self-harm. Crisis. (2011) 32:74–80. doi: 10.1027/0227-5910/a000060, PMID: 21616756

[26] Lloyd-Richardson EE Perrine N Dierker L Kelley ML. Characteristics and functions of non-suicidal self-injury in a community sample of adolescents. Psychol Med. (2007) 37:1183–92. doi: 10.1017/S003329170700027X, PMID: 17349105 PMC2538378

[27] Reinhardt M Kökönyei G Rice KG Drubina B Urbán R. Functions of nonsuicidal self-injury in a Hungarian community adolescent sample: a psychometric investigation. BMC Psychiatry. (2021) 21:618. doi: 10.1186/s12888-021-03613-4, PMID: 34886827 PMC8662905

[28] Thai TT Jones MK Nguyen TP Pham TV Bui HHT Kim LX. The prevalence, correlates and functions of non-suicidal self-injury in Vietnamese adolescents. Psychol Res Behav Manag. (2021) 14:1915–27. doi: 10.2147/PRBM.S339168, PMID: 34866944 PMC8636691

[29] Yen S Kuehn K Melvin C Lauren M Weinstock LM Andover MS. Predicting persistence of nonsuicidal self-injury in suicidal adolescents. Suicide Life Threat Behav. (2016) 46:13–22. doi: 10.1111/sltb.12167, PMID: 25907682 PMC4619186

[30] Tabachnick BG Fidell LS. Using multivariate statistics. 5th ed. Boston: Allyn & Bacon/Pearson Education (2001).

[31] Muehlenkamp JJ Brausch AM Washburn JJ. How much is enough? Examining frequency criteria for NSSI disorder in adolescent inpatients. J Consult Clin Psychol. (2017) 85:611–9. doi: 10.1037/ccp0000209, PMID: 28414487 PMC5461948

[32] Wolff JC Thompson E Thomas SA Nesi J Bettis AH Ransford B. Emotion dysregulation and non-suicidal self-injury: a systematic review and meta-analysis. Eur Psychiatry. (2019) 59:25–36. doi: 10.1016/j.eurpsy.2019.03.004 30986729 PMC6538442

[33] Gratz KL. Risk factors for and functions of deliberate self-harm: an empirical and conceptual review. Clin Psychol Sci Pract. (2003) 10:192–205. doi: 10.1093/clipsy.bpg022

[34] Edmondson AJ Brennan CA House AO. Non-suicidal reasons for self-harm: a systematic review of self-reported accounts. J Affect Disord. (2016) 191:109–17. doi: 10.1016/j.jad.2015.11.043, PMID: 26655120

[35] Briere J Eadie EM. Compensatory self-injury: posttraumatic stress, depression, and the role of dissociation. Psychol Trauma Theory Res Pract Policy. (2016) 8:618–25. doi: 10.1037/tra0000139, PMID: 27077490

[36] Franklin JC Puzia ME Lee KM Lee GE Hanna EK Spring VL. The nature of pain offset relief in nonsuicidal self-injury. Clin Psychol Sci. (2013) 1:110–9. doi: 10.1177/2167702612474440

[37] Selby EA Nock MK Kranzler A. How does self-injury feel? Examining automatic positive reinforcement in adolescent self-injurers with experience sampling. Psychiatry Res. (2014) 215:417–23. doi: 10.1016/j.psychres.2013.12.005, PMID: 24388504

[38] Ong SH Tan ACY Liang WZ. Functions of nonsuicidal self-injury in Singapore adolescents: implications for intervention. Asian J Psychiatr. (2017) 28:47–50. doi: 10.1016/j.ajp.2017.03.015, PMID: 28784396

[39] Chapman AL Gratz KL Brown MZ. Solving the puzzle of deliberate self-harm: the experiential avoidance model. Behav Res Ther. (2006) 44:371–94. doi: 10.1016/j.brat.2005.03.005, PMID: 16446150

[40] Allen C. Helping with deliberate self-harm: some practical guidelines. Ment Health. (1995) 4:243–50. doi: 10.1080/09638239550037523

[41] Nock MK Wedig MM Holmberg EB Hooley JM. The emotion reactivity scale: development, evaluation, and relation to self-injurious thoughts and behaviors. Behav Ther. (2008) 39:107–16. doi: 10.1016/j.beth.2007.05.005, PMID: 18502244

[42] Turner BJ Cobb RJ Gratz K Chapman AL. The role of interpersonal conflict and perceived social support in nonsuicidal self-injury in daily life. J Abnorm Psychol. (2016) 125:288–98., PMID: 26845256 10.1037/abn0000141 PMC5493473

[43] Victor SE Muehlenkamp JJ Hayes NA Lengel GJ Styer DM Washburn JJ. Characterizing gender differences in nonsuicidal self-injury: evidence from a large clinical sample of adolescents and adults. Compr Psychiatry. (2018) 82:53–60. doi: 10.1016/j.comppsych.2018.01.009, PMID: 29407359 PMC5845831

[44] Crick NR Zahn-Waxler C. The development of psychopathology in females and males: current progress and future challenges. Dev Psychopathol. (2003) 15:719–42. doi: 10.1017.S095457940300035X, PMID: 14582938

[45] Gardner KJ Paul E Selby EA Klonsky ED Mars B. Intrapersonal and interpersonal functions as pathways to future self-harm repetition and suicide attempts. Front Psychol. (2021) 12:688472. doi: 10.3389/fpsyg.2021.688472, PMID: 34349705 PMC8326376

[46] Reinhardt M Rice KG Horváth Z. Non-suicidal self-injury motivations in the light of self-harm severity indicators and psychopathology in a clinical adolescent sample. Front Psychiatry. (2022) 13:1046576. doi: 10.3389/fpsyt.2022.1046576, PMID: 36532173 PMC9751932

[47] Brausch AM Muehlenkamp JJ. Perceived effectiveness of NSSI in achieving functions on severity and suicide risk. Psychiatry Res. (2018) 265:144–50. doi: 10.1016/j.psychres.2018.04.038, PMID: 29709788 PMC5984167

[48] Schoenleber M Berenbaum H Motl R. Shame-related functions of and motivations for self-injurious behavior. Personal Disord Theory Res Treat. (2014) 5:204–11. doi: 10.1037/per0000035, PMID: 24364497

[49] Oppenheimer CW Glenn CR Miller AB. Future directions in suicide and self-injury revisited: integrating a developmental psychopathology perspective. J Clin Child Adolesc Psychol. (2022) 51:242–60. doi: 10.1080/15374416.2022.2051526, PMID: 35380885 PMC9840868

[50] May AM Pachkowski MC Klonsky ED. Motivations for suicide: converging evidence from clinical and community samples. J Psychiatr Res. (2020) 123:171–7. doi: 10.1016/j.jpsychires.2020.02.010 32078834

[51] Linehan M. Cognitive-behavioral treatment of borderline personality disorder. New York: Guilford Press (1993).

[52] Huguet A Rao S McGrath PJ Wozney L Wheaton M Conrod J. A systematic review of cognitive behavioral therapy and behavioral activation apps for depression. PLoS One. (2016) 11:e0154248. doi: 10.1371/journal.pone.0154248, PMID: 27135410 PMC4852920

[53] Zerubavel N Messman-Moore TL. Staying present: incorporating mindfulness into therapy for dissociation. Mindfulness. (2013) 6:303–14. doi: 10.1007/s12671-013-0261-3

